# Pandemic (H1N1) 2009 and Oseltamivir Resistance in Hematology/Oncology Patients

**DOI:** 10.3201/eid1611.101053

**Published:** 2010-11

**Authors:** Cameron Wolfe, Ian Greenwald, Luke Chen

**Affiliations:** Author affiliation: Duke University Medical Center, Durham, North Carolina, USA

**Keywords:** Influenza, influenza A, pandemic (H1N1) 2009, viruses, oseltamivir, antimicrobial resistance, hematologic neoplasms, oncology, letter

**To the Editor:** Tramontana et al. (*1*) recently described characteristics and oseltamivir resistance in hematology and oncology patients infected with pandemic (H1N1) 2009 virus. Such cases merit further study because concurrent medical problems in immunosuppressed patients may obscure and delay diagnosis and management of pandemic (H1N1) 2009 infections. Moreover, severe complications of such infection may be more likely to develop in immunosuppressed patients (*2*). During the winter of 2009, oseltamivir-resistant pandemic (H1N1) 2009 virus infection was diagnosed for 4 patients at Duke University Medical Center. We describe the clinical features of the infections, the challenges associated with diagnosis of pandemic (H1N1) 2009 virus infection, and the clinical outcome for the infected patients.

Four immunocompromised patients who received chemotherapy and immunotherapy for solid-organ and hematologic malignancies were hospitalized at our tertiary care medical center during October–November 2009, a period of peak activity of pandemic (H1N1) 2009 in surrounding communities in North Carolina (*3*). These 4 case-patients experienced symptoms attributable to pandemic (H1N1) 2009 from 0 to 14 days after hospital admission, and the diagnosis of pandemic (H1N1) 2009 was made 0–28 days after symptom onset. Illness, diagnosis, and treatment of the patients are summarized in the Table. One patient reported contact with a family member who had influenza-like illness. Three other patients likely acquired pandemic (H1N1) 2009 in the hospital. An investigation could not conclusively establish whether transmission of pandemic (H1N1) 2009 occurred between case-patients and healthcare workers or visitors (*4*). All 4 case-patients ultimately died; 2 patients recovered from pandemic (H1N1) 2009 after antiviral drug therapy but died of underlying disease and subsequent bacterial infections. One case-patient did not receive antiviral drugs because the diagnosis was made posthumously.

**Table:** Clinical, diagnostic, and therapeutic patient information for 4 patients hospitalized for hematologic and oncologic conditions, North Carolina, USA, 2009*

Patient information	Patient no./age, y/sex			
	1/43/F	2/58/F	3/67/F	4/61/M
Underlying disease	Relapsed acute myelogenous leukemia	Refractory mycosis fungoides	Recurrent metastatic thymoma	B-cell acute lyphoblastic lymphoma
Reason for admission	Scheduled consolidative chemotherapy	Staphylococcal sepsis; recent interferon-α; malnutrition	Progressive fevers and hypoxia; diffuse; infiltrates shown on chest radiograph	Fevers and respiratory compromise at home, after recent chemotherapy
Signs/symptoms on admission	Intermittent fever during early admission; d 14† cough, persistent fevers; d 24 progressive hypoxia	Intermittent fevers; d 27, cough; persistent fevers, progressive hypoxia	Fever for 5 d; hypoxia, widespread pulmonary infiltrates	Daily fevers for 5 d, cough, hypoxia, fatigue, generalized weakness; diffuse infiltrates on radiograph
Use of oseltamivir	Yes, for 10 d; 75 mg daily prophylaxis after known exposure 2 d prior	No	No	No
Diagnostic information	d 14, nasal wash PCR negative‡; d 25, BAL positive for pandemic (H1N1) 2009 virus; d 37, BAL remained positive, H275Y mutation	d 27 nasal wash positive for pandemic (H1N1) 2009 virus; d 44, H275Y mutation confirmed on d 27 specimen; medication modified	d 1, nasal wash positive for pandemic (H1N1) 2009 virus; d 4, d 9, bronchoscopy results positive; d 12, bronchoscopy results negative for influenza on culture and PCR; H275Y mutation detected on all positive specimens§	d 23 bronchoscopy and d 27 nasal wash results negative; d 28 bronchoscopic viral culture positive for pandemic (H1N1) 2009 virus, 1 d after patient’s death; H275Y mutation detected by CDC
Factors confounding pandemic (H1N1) 2009 diagnosis	Consolidation chemotherapy; *Escherichia coli* bacteremia, presumptive fungal pneumonia, persistent leukopenia neutropenia postchemotherapy	*Staphylococcus aureus, Pseudomonas aeruginosa, Klebsiella pneumoniae* bacteremia; recent interferon-α therapy; salvage chemotherapy; persistent leukopenia and neutropenia at discharge	Recent thoracic radiotherapy; catheter-associated *Staphylococcus aureus* bloodstream infection.	Consolidative chemotherapy with prolonged neutropenia; significant emphysema; catheter-associated pseudomonal bloodstream infection
Treatment	Oseltamivir,75 mg 2×/d for 5 d; then, 150 mg 2×/d until death; mechanical ventilation for 15 d	Oseltamivir, 75 mg 2×/d for 9 d; then 150 mg 2×/d for 8 d; modified to renally adjusted IV zanamivir for 10 d, when mutation detected	Oseltamivir, 75 mg 2×/d for 12 d; mechanical ventilation for 16 d	Broad-spectrum antibacterial agents; antiviral agents (not influenza agents), and antifungal agents; mechanical ventilation for 48 h
Clinical outcome	Died 38 d postadmission; refractory respiratory failure and progressive ARDS	Improvement in respiratory status following zanamivir treatment; ultimately, failure of bone marrow recovery; died 4 d after discharge to hospice	Refractory respiratory failure; septic shock; decision to withdraw care	Refractory respiratory failure and elective withdrawal of care

*BAL, bronchoalveolar lavage; ARDS, acute respiratory disease syndrome; IV, intravenous; CDC, Centers for Disease Control and Prevention. †Days postadmission. ‡All specimens, unless otherwise stated, were tested with proFlu Plus PCR (Prodesse, Waukesha, WI, USA) for influenza viruses A and B and respiratory syncytial virus. No quantitative tests were available. §Mutation genotype confirmed at CDC. Results were available posthumously for patients 1, 3, and 4. No mutation that conferred zanamivir resistance was detected.

We learned valuable lessons regarding diagnosis and management of pandemic (H1N1) 2009 in immunocompromised patients. First, pandemic (H1N1) 2009 infection can be difficult to diagnose in immunocompromised hospitalized patients. Such patients do not exhibit consistent symptoms or signs for pandemic (H1N1) 2009. Consistent with Tramontana et al. (*1*), fever was the most common feature, followed by progressive dyspnea and intermittent cough. None of our patients reported sore throat. Moreover, such nonspecific symptoms may be inadvertently attributed to concurrent medical problems common in immunocompromised patients such as bone marrow suppression, adverse effects of drugs or chemotherapy, recent surgical procedures, opportunistic infections, or line-related bloodstream infections.

Second, respiratory viruses may be imported and subsequently transmitted to hospitalized patients despite standard infection prevention measures (*5*). Clinicians should remain vigilant for hospital-onset respiratory viral infections and have a low threshold for diagnostic testing, particularly during periods of increased influenza or respiratory virus activity in the community.

Early suspicion and prompt testing may have reduced the delay in the diagnosis and management of these patients with pandemic (H1N1) 2009. However, the initial nasal wash specimen from patient 1 was negative for pandemic (H1N1) 2009 virus antigen, whereas the initial bronchoscopy and nasal wash specimens from patient 4 also were negative for such antigens on laboratory testing. This underscores the limitations of current testing and that the sensitivity of pandemic (H1N1) 2009 diagnostic testing remains poor and needs further improvement. Thus, in patients suspected of having pandemic (H1N1) 2009 or in those who are critically ill, lower tract respiratory specimens should tested to improve diagnostic sensitivity, and clinicians should consider using immunoassay and culture methods.

All 4 patients described in this case series had viral isolates containing H275Y mutation in the neuraminidase gene of pandemic (H1N1) 2009 virus, which is specifically associated with high-level resistance to oseltamivir. Increasing data show that immunocompromised patients are at increased risk for development of drug-resistant influenza infections after oseltamivir prophylaxis or while receiving oseltamivir treatment (*6*). Fortunately, this resistance trait remains rare.

Existing evidence suggests oseltamivir-resistant pandemic (H1N1) 2009 virus is stable and retains similar transmissibility and virulence as the wild-type virus (*7*). Therefore, in immunosuppressed patients, in which the influenza mortality rate is high, clinicians should also suspect drug-resistant influenza infection if the patient does not improve. Before she died of underlying hematologic illness, patient 2 clinically improved after treatment with intravenous zanamivir (obtained through an emergency application for an investigational–new drug). As reported in other studies, pandemic (H1N1) 2009 virus was found in her nasal washes, 1 week after she received zanamivir for 10 days (*8*).

Some data suggest that pandemic (H1N1) 2009 virus has a predilection to affect the lower respiratory tract and is associated with more illness and death than is seasonal influenza (*9*). All 4 case-patients with in this series developed dyspnea, and 3 of the 4 ultimately died of refractory respiratory failure. Our observations suggest that oseltamivir-resistant pandemic (H1N1) 2009 virus is also associated with poor prognosis and may retain the same tropism for lower respiratory tract involvement as wild type.

These case-patients illustrated the complexity of the diagnosis and management of such infections in hospitalized immunocompromised patients. Vigilance and heightened clinical suspicion are needed to facilitate early diagnosis, treatment and prevention measures to limit transmission of pandemic (H1N1) 2009 virus or similar viral pathogens.
